# Resisting technological inevitability: Google Wing’s delivery drones and the fight for our skies

**DOI:** 10.1098/rsta.2024.0107

**Published:** 2024-11-13

**Authors:** Anna Zenz, Julia Powles

**Affiliations:** ^1^ UWA Tech & Policy Lab, Law School, The University of Western Australia, 35 Stirling Highway, Crawley, WA 6009, Australia; ^2^ Digital Civil Society Lab, Stanford University, 559 Nathan Abbott Way, Stanford, CA 94305, USA; ^3^ Centre for Business Research, University of Cambridge, 11-12 Trumpington Street, Cambridge CB2 1QA, UK

**Keywords:** drones, Google, inevitability, tech determinism, smart cities, tech resistance

## Abstract

Efforts to realize on-demand delivery drone networks present a stark example of how the technology industry seeks to dominate new markets, regardless of societal consequences. Analyzing the most advanced of these efforts—Google Wing’s operations in Australia since 2017—we identify the instrumental role of narratives of technological inevitability (of tech expansion, and societal adaptation) in catalyzing new sky-based commerce. Yet the interest of this case study lies in a twist. Google Wing’s rollout in Australia’s capital, Canberra, initially proceeded as a textbook example of tech expansion. However, citizen engagement and public governance dramatically intervened and, we argue, disrupted the logic of technological inevitability. This article is the first to analyze these dynamics, many of which originated with Bonython Against Drones (BAD), a community action group forged from those who first lived under Google’s food delivery drones. The article exposes the flawed logic of technological inevitability as the enabling force of tech expansion; characterizes the governance failures that help install corporate visions for public goods; animates the potentialities of communities living with new technologies; and identifies the sky itself, as both a public commons and a vital, living habitat, as a key future locus for participatory governance.

This article is part of the theme issue ‘Co-creating the future: participatory cities and digital governance’.

## 1. Introduction

### (a) Google moves into the city

Since the mid-2000s, cities have been key targets for technology companies seeking to create and dominate new markets [1–3]. Few companies have been bolder in pursuing the city as a valuable digital and physical testing ground than Google LLC and its parent company, Alphabet Inc. [4–8]. Even before launching its infamous ‘urban innovation’ arm Sidewalk Labs in 2015 [6–8], Google had initiated an audacious claim to cities and skies globally: its delivery drone venture.

‘Project Wing’ was established in 2012 under X, Google’s ‘laboratory for chasing moonshot ideas’ [9]. Its declared mission was ‘building delivery drones, and working towards the day when these aircraft can deliver everything from consumer goods to emergency medicine—a new commerce system that opens up universal access to the sky’ [10,11]. In 2018, the project graduated from X to become an independent business of Alphabet, Wing Aviation LLC [12]. Over time, Wing has established limited operations in Australia (2017) [13], Finland (2019) [14], the United States (2019) [15] and Ireland (2022) [16].

The core business of Google Wing (a naming convention used to reinforce corporate lineage) is providing on-demand, app-based drone delivery of generally single-item consumer and household goods; predominantly, hot and cold drinks (led by flying cups of coffee [17,18]), snacks, meals and, to a lesser extent, grocery and pharmacy products [19]. Wing has delivered these items at a modest scale in select suburbs in its four countries of operation, through economics made possible by Google’s enormous reserves of patient capital [20]. In proving its service, Google Wing absorbed all costs of delivery, with consumers paying merchants only the standard price of purchased items [21]. Since 2019, Wing has also been developing a broader logistics capability through its Uncrewed Traffic Management system [22,23] and Wing Delivery Network [24]. Comprising integrated platform services, such as Wing’s ‘OpenSky’ flight management software [25] and ‘AutoLoader’ curb-side pick-up [24], these systems and accompanying global lobbying efforts [23] presage Google Wing’s long-term vision to be at the centre of managed aerial delivery and transit.

Australia is home to Google’s most substantial drone operation, both by volume of deliveries and customers served [26–28]. Indeed, of the 100 000 deliveries Google Wing made in 2021, more than 50 000 were to customers in Logan, Queensland (Wing’s second city after its initial launch in Canberra), prompting the company to crown Logan the ‘drone delivery capital of the world’ [19]. Australian governments at all levels have welcomed and capitalized on this reputation. At the federal level, this has involved a swift increase in policies to grow the commercial drone industry, commencing in earnest in 2019 [29–31]. At the same time, states [32,33], territories [34] and cities [35] have sought to attract Google and other drone operators, making direct appeals in the name of boosting economic growth and innovation [36].

Wing’s ostensible success story in Australia must be contrasted with the experiences of communities living under skies thrumming with delivery drones. As this article examines in depth, Google’s 2018 introduction of delivery drones to its first residential test-site, the small southern Canberra suburb of Bonython, was plagued by significant community backlash against a litany of detrimental impacts that drones impose on residents and the environment, as well as severe failures in governance and oversight. This led to a 2019 Parliamentary Inquiry [37], sustained media attention [38–40], regulatory reform [41] and, in 2023, Wing ultimately abandoning its operations in Canberra and the Australian Capital Territory (ACT) [42].

Wing’s initial venture into Australian cities and airspace parallels many of the dynamics of other Google-affiliated companies expanding into new domains [6,43,44], and illustrates the vital importance of political and community conduits in either facilitating or resisting this expansion [8]. Previous studies have critically assessed the merits of ‘smart city’ projects, their potential pitfalls and the companies behind them [6,44–49], as well as the role of narratives in installing dominant sociotechnical imaginaries [50,51]. This article contributes to this literature by illuminating a particularly striking example of what Sadowski and Bendor have termed ‘corporate discourses as tools for directing and delimiting what we can imagine as possible’ [46]. Using the central case study of Google Wing’s first residential delivery drone trials globally, we examine narratives of technological inevitability as a tool of corporate and political power, and the material consequences that follow when these narratives are accepted, replicated or resisted.

### (b) Conceptual framework: narratives of technological inevitability

Technological inevitability lies in the ideological tradition of *technological determinism*, which broadly denotes belief in the deterministic nature of technology and its effects, that ‘technological change determines social change in a prescribed manner’ [52]. ‘Technology’ in this context is broad, spanning from technological artefacts and abstractions to sociotechnical systems. In recognition of the multiple schools of thought in the tech-determinism literature, we prefer the term *technological inevitability*, for two reasons. First, depending on where a particular theory is located on the spectrum between what some scholars call ‘hard’ and ‘soft’ tech-determinism, technology may be treated as an exclusive agent of change [53,54], or social factors may also be credited as affecting technology and its consequences [55]. By contrast, the notion of ‘inevitability’ is absolute. It denies any possibility of human influence over what are cast as natural, self-fulfilling ‘technological forces that operate beyond human agency and the choices of communities’ [4]. Second, the construct of inevitability most aptly captures the dynamics and narratives explored in this article: the systemic disempowerment of governments and communities, in the face of the stories they are told (and, in turn, retell) about the unstoppable arrival and expansion of new technology.

Our contribution adds to scholarship showing how technological determinism and its variants—including techno-solutionism [56], tech-chauvinism [57] and tech-fatalism [58]—conceal economic, political and social reconfigurations of power that attend tech expansion. We also build on research demonstrating how tech companies utilize narratives to legitimize self-serving versions of the future [59,60], including specifically in the context of drones [61–64]. In emphasizing narratives of technological inevitability, we draw particular inspiration from Zuboff’s *The Age of Surveillance Capitalism*, which identifies inevitability rhetoric as so pervasive within the tech industry that it constitutes ‘a full-blown ideology of inevitabilism’ [4]. Writing about the Silicon Valley ambition ‘that *everything* will be connected, knowable, and actionable’, Zuboff describes how the ‘doctrine of inevitability’ both deftly conflates ‘commercial imperatives and technological necessity’ and is programmed to ‘delete resistance and creativity from the text of human possibility … render[ing] us helpless and passive in the face of implacable forces’ [4]. As Sacasas explains, similarly: ‘Narratives of inevitability have the effect of foreclosing thought and deliberation. If outcomes are inevitable, then there’s nothing to do but assimilate to this pre-determined future, to go along for the ride prepared for us whatever the consequences’ [65]. Ultimately, inevitability narratives eviscerate opportunities for refusal, described by Gangadharan as ‘agentic possibilities of willful self-exclusion’ [66].

A focus on narratives helps showcase the many flexible guises of inevitability rhetoric. Narratives of ever-expanding technological development can be optimistic, pessimistic and everything in between, across an endless array of technologies—the cloud, metaverse, artificial intelligence, drones, self-driving cars, wearables and more. The unifying factor is displacement from the action: technology presents its own drama, and we are all onlookers. We can be enthused, resigned or concerned about technology and how it affects society—and we can act on these responses. But the narrative and its direction of travel are presented as untouchable. Under the logic of inevitability, we cannot intervene or change the drama, like time-travellers from another timeline.

This article examines two key objectives of Google Wing in deploying inevitability narratives in the delivery drone context, each of which has broader resonances across the tech industry. The first is *political enablement*: government actors who believe that drones will inevitably feature throughout urban environments, for example, will see benefit in embracing this certainty sooner rather than later. Narratives of inevitability therefore encourage government actors to surrender their power and to offer political support, either passively or actively, to tech companies. This facilitates tech expansion through the political process [67]. The second aim is *community acquiescence*: if the public anticipates that delivery drones will inevitably fill the sky, they are more likely to be silent or tolerant about this prospect, both of which can be taken as signaling acceptance. Here the logic of inevitability works to render resistance futile and incapacitate the public, facilitating tech expansion by removing the need for public approval.

### (c) Methodological approach

This article examines the circulation of narratives of the inevitability of delivery drones in the founding years of Google Wing’s drone operations and traces the effectiveness of, and disruptions to, these narratives. We focus first on Google’s corporate narratives and their acceptance and replication by government actors, as a mode of analyzing what Fuchs and Kalfagianni have coined the discursive power of corporations: ‘the capacity to influence policies and political processes through the shaping of norms and ideas’ [68]. This is accompanied by a detailed case study of the strategies and narratives of the community action group, Bonython Against Drones (BAD). Inevitability narratives are rarely discussed or critically evaluated [4], and we found that in most cases they are subtle and beneath the surface—unseen and unquestioned until unearthed and centred. This makes the case study particularly productive, since centring and challenging inevitability rhetoric was a key feature of BAD’s counter-narrative.

To achieve its research aims, the article is based on a close reading of source material about Google Wing and its Australian drone operations, including media statements, public presentations, speeches, interviews, websites, blogs, marketing material, news coverage, policy submissions, ministerial statements, parliamentary evidence and reports, and correspondence between Google Wing, government actors and community members. These materials were identified through targeted web searches and informed by field visits and meetings with key actors in Logan, Queensland (November 2022) and Canberra, ACT (February 2023), along with sustained engagement with Australian drone policy developments by UWA Tech & Policy Lab researchers since October 2020 [69–71].

The study utilizes extensive sources of evidence on Google Wing’s Bonython trial that, while publicly available, have not been subject to scholarly analysis. In particular, we draw on 1119 pages of primary source documents that were released in March and April 2019 by two ACT Government Directorates (Chief Minister, Treasury and Economic Development; and Environment, Planning and Sustainable Development) in response to *Freedom of Information Act 2016* (ACT) requests from community members in Bonython as part of a meticulous grassroots campaign [72–80]. These documents provide extraordinary historic detail on the Bonython trial and are further complemented by submissions, transcripts and reports from the 2019 Legislative Assembly Inquiry into Drone Delivery Systems in the ACT (‘Parliamentary Inquiry’).

The source material was reviewed to identify and characterize recursive narrative forms, of which the most common were narratives of technological inevitability, as described in this article. Consistent with the article’s objective of explicating the nature and power of these narratives—and how they may be, and have been, resisted—the surveyed material provides a solid evidentiary basis from which to develop an interpretative analytical account.

The sections that follow provide illustrative evidence to support our account of how inevitability narratives have been mobilized in Wing’s drone venture. Section 2 examines how Google Wing made Australia its first global test-site for delivery drone operations, and identifies and analyzes the corporate narratives instrumental to this endeavour. Section 3 demonstrates how ACT Government actors politically enabled Wing’s operations through repeatedly accepting and replicating corporate narratives that elided responsibility for public governance and oversight. Section 4 elaborates the case study of BAD, demonstrating how this small and unlikely community action group developed a compelling and sustained counter-narrative of resistance to Google Wing’s delivery drones. Section 5 concludes with lessons on the flawed logic of inevitability, the aftermath of resistance, and how Google Wing adapted its service in moving from the ACT to Queensland. Given that narratives of technological inevitability are inimical to human agency and political participation, critical engagement with their role as a discursive practice [81] presents a particularly urgent challenge to the role of digital technologies in participatory governance [82].

## 2. Google drones in Australian skies

### (a) ‘Selecting’ Canberra

Californian-headquartered Wing was established to develop ‘fast and efficient local delivery options’ to challenge road-based transit [13]. After five years of research and development, including isolated test flights on Queensland farms [83,84] and at Virginia Tech’s campus [85], by 2017 the company’s lead test-site had become Australia. The story of how Wing ended up in Australia has never been fully told, though with 20/20 hindsight both sides have presented it as an inevitable match.

Wing’s Australian operations commenced with three discrete, geographically limited trials close to Australia’s political heartland: Googong, NSW, in July 2017 [86]; Royalla, near the border of NSW and the ACT, from September 2017 to March 2018 [87]; and Bonython, Canberra, from July 2018 to February 2019 [87]. The most notable of these was Bonython, given it was the company’s first delivery trial in a residential, suburban setting, and became the site of its most sustained controversy and resistance.

From the beginning, a cornerstone of Google Wing’s political narrative strategy was the flattering portrayal of Australia, and particularly its capital, Canberra, for having ‘staked out a leadership position in the advancement of drone technology’ [88]. Given Canberra has the rare distinction of being the seat of three levels of government—federal (Commonwealth of Australia), territorial (ACT) and local (City of Canberra, through the ACT Government)—Google Wing’s focus on the city and its political class proved efficient and astute. In correspondence with key ACT ministers, Chief Minister Andrew Barr MLA and Mick Gentleman MLA, Wing repeatedly emphasized the reputation of Australia’s federal Civil Aviation Safety Authority (CASA) (also Canberra-based) as ‘one of the most innovative aviation regulators in the world’ [13]. In public messaging, Wing similarly lauded CASA for ‘leading the way’ [21] with its ‘progressive’ approach [89]. Invariably, Wing also marketed how Australia offered ‘an interested consumer population, a talented and experienced unmanned aviation workforce, and enthusiastic and innovative business and government partners’ [88].

Google Wing identified and carefully encouraged a receptive audience within the ACT Government by highlighting the ACT’s ambition to be ‘a hub for trialling new ideas’ [35], and Canberra as ‘a city that engages productively with innovation and research’, with associated ‘reputational advantages’ [87]. Wing noted ‘the ACT Government’s commitment to evolving Canberra into a smart and connected digital city and Canberrans’ enthusiasm as early adopters of new technology’ [13]. Readily adopting this image, ACT Government actors built support for Google Wing’s ACT presence (discussed further in §3) around the central theme of ‘innovation’—as a mindset, a goal and a conduit for myriad economic and social benefits [87]. Two defining pillars of this image of the ACT were the *selection* of Canberra [35], instilling a sense of competition and triumphalism that has proven effective in Google’s previous advances to city administrations [6], and Canberra’s place as Wing’s *first* residential test-site, taken as evidencing the city’s ‘growing reputation as a progressive regulatory environment that is business-friendly and helping to foster innovation’ [90].

### (b) Situating drones as inevitable

A major finding of our analysis of Google Wing’s operations in Australia is that the *inevitability of drones* served as the foundational logic, or propulsive force, of all aspects of corporate and political discourse. While not presented in the direct terms ‘drones are inevitable’, this was the inexorable and overriding effect of presenting drones through one or more of the narrative lenses we describe as *narratives of necessity*, *desire* or *presumption*. All three slot elegantly into how Wing primed the city of Canberra for its drone trials.

*Narratives of necessity* or *desire* intone ‘drones are coming’ and appeal to the *need* or, alternatively, *desire* to ‘get on board or get left behind’ [65]. Promises of economic growth drive these narratives, along with an assortment of necessary (and sometimes urgent) or desirable ends—targeted appeals to how drones will address urban policy priorities, such as reducing traffic congestion, emissions and accidents (all premised on the contestable notion that drone flights replace road traffic); assist accessibility; combat urban sprawl; and drive abstract goals like ‘agility’ and ‘innovation’. These appeals are representative of corporate narratives of smart urbanism that portray technological solutions as the inevitable response to impending urban crises [46]. An example of a narrative that combines necessity (‘now, now, now’) with desire (‘economic benefits’, ‘agile’ and ‘innovative’) is this excerpt from an October 2017 ACT Government discussion paper:

‘The use of drone for delivery is now emerging as a serious business model … Jurisdictions are now starting to seriously consider the matter … With Project Wing’s introduction, we are now being prompted to consider our own position on delivery drones … Apart from the direct economic benefits from Project Wing’s investment in Canberra, Project Wing’s expansion in Canberra aligns with the ACT’s business development strategy and has the potential to strengthen the ACT’s reputation as an agile and innovative jurisdiction.’ [91]

In *narratives of presumption*, the message is ‘drones are here’ and, more than that, they are ubiquitous. Foregone are questions of *why* (as in the necessity/desire narratives). Instead, the narrative focus shifts to *what next*, particularly in terms of how to accommodate drones at scale. Driving this narrative is an appeal to participate in drone futures, despite this participation being passive in practice. Google Wing makes this narrative explicit in pitching its vision of a holistic Uncrewed Traffic Management system:

‘The future of aviation is here, and it’s much smaller, more widespread and numerous than we could have ever imagined. While it may feel like this is a far-off, science-fiction future, it’s not … The question now is not if drones will be a part of our future; it’s how will we manage this future that is already here?’ [25]

Interestingly, we found each of these narratives to be highly effective, independent of actual community responses to drones. We posit that a large part of their strength as corporate and political discourses comes from implying a *collective* sentiment of necessity, desire or presumption regarding the ‘inevitable’ rise of delivery drones—a sentiment that overrides the responses of any individual or group.

### (c) Managing community ‘acceptance’

Delivery drones have many distinctive features that affect communities. Most significantly, they create new routes of sky-based commerce, where none previously existed. These routes are visibly and audibly intrusive on resident populations [92], as well as on birds, insects and mammals in the living habitat of the skies. Recognizing the very small number of people using the service relative to the affected community, a Wing representative stated in December 2023, ‘We fly over hundreds of thousands of people every day [to do] thousands of packages of delivery every week’ [93]. In the Bonython trial, access to the service was restricted to 164 ‘testers’ out of approximately 4000 residents, meaning a service used by less than 5% of the local population had to be endured by the other 95% [88,94]. Recalling the items that drones deliver (drinks, fast food, that one forgotten item from the shopping list), this is a considerable imposition on the population at large for the convenience of a small minority. How was this pitched to communities?

Google Wing’s community engagement approach mirrored the same narratives of necessity, desire and presumption used politically, with the company taking the position that adoption ‘depends on customers and communities seeing the value of the new service provided and embracing rather than resisting new ways to receive products’ [92]. The principal community value Wing presented was ‘the potential to save businesses and consumers in Australia time and money, while also helping to reduce congestion, greenhouse gas emissions and accidents on the road’ [95]. Again, this relies on the unproven premise that drones replace road traffic, while obscuring that they necessitate the creation of new commercial highways in the commons of the sky. Implicitly, Wing is making a bet on the continued growth of the food delivery market (often referred to by the US phrase ‘last-mile delivery’), which surged during the COVID-19 pandemic. Studies have shown that food delivery apps such as Uber Eats and DoorDash, which are tremendously popular in Australia, are successful in part because they prioritize individual behavior, separating customers from the realities of these services for workers [96,97], and certainly from broader public health [98] and environmental costs.

A feature of Wing’s narrative approach is the recurring conflation of what we term ‘indulgence’ or ‘convenience’ versus ‘necessity’ applications for drones [36,69–71], through promoting drone use ‘[w]hether you’re a parent with a sick child at home and have run out of baby paracetamol … or you simply just want to order your morning flat white without the hassle of having to drive to the café’ [99]. Invoking societal interest in medical care as a critical component of the drone convenience equation is a deliberate rhetorical strategy replicated by political actors [69,100,101]. The strategy is informed by research showing that public acceptance of drones increases for applications of drones perceived to be of higher societal value [102–104]. Reflecting this approach, and despite knowledge that single cups of coffee had persisted as Wing’s leading delivery item, the ACT Government cited the potential for drones to deliver pharmaceutical supplies to people living with disabilities as a post-hoc justification for supporting Google Wing’s trials [87].

Wing’s community engagement strategy emphasizes the importance of ‘capturing public sentiment’ and ‘gauging how to grow and adapt’ through community feedback [92]. However, in practice, the firm’s engagement is strikingly one-sided. Relying on Google’s public relations expertise, Wing is adept at building community *awareness* through demonstrations, pop-ups, town halls, marketing campaigns and local partnerships [88,105]. Yet when it comes to community *feedback*, and particularly negative feedback, its approach is starkly numeric. Starting in Canberra, Wing seeded a dangerous precedent, since normalized by government actors: relying on the bald number of complaints as the sole metric of ‘community acceptance’ of drones [106]. This approach is deeply flawed, since complaints represent only the most aggrieved and motivated critics and, even in those cases, complaint mechanisms must be accessible and trustworthy, generally through independence. Despite the evident shortcomings of Wing’s approach, government actors have been loath to get involved, even when Wing has mischaracterized community responses. This is pertinent in relation to Bonython, where Wing has pointedly sought to erase the controversy and sustained criticism it attracted (see §4) from its corporate record [99]. Implicitly affirming this approach, ACT Chief Minister Barr wrote to an aggrieved resident following the conclusion of the trial: ‘How Project Wing wish to present the outcomes of the Bonython trial and its engagements with community is their business decision’ [107]. Unpacking how the ACT Government came to this position is the task of the next section.

## 3. Google Wing’s political enablement

This section analyzes the political backdrop to Wing’s first residential drone operations, demonstrating how the ACT Government steadily abandoned its political will to govern Wing’s trials. We attribute this failure to deference to corporate narratives, leading to an avoidance of jurisdictional authority and responsibility.

### (a) Corporate deference and governance failure

When Google Wing launched in Australia, it took full advantage of the enabling regulatory framework for drones, administered by CASA [108,109]. In 2001, CASA was the first national aviation authority globally to adopt drone-specific legislation [102]. The regime it developed has two aims: to manage drone safety risks, while at the same time not discouraging ‘innovation or impos[ing] unnecessary regulatory costs on either commercial operators or recreational users’ [110,111]. Its backbone is a system of default safety rules with allowances for exemptions [112,113]. To conduct its trials, Wing secured exemptions from CASA to operate its delivery drones near people, over populous areas and beyond visual line of sight—all of which the default rules would otherwise prohibit [114,115].

While CASA’s regulatory framework adequately addresses the *physical safety* risks posed by drones, it is silent on other regulatory concerns including noise, visual pollution, privacy, security and disturbances to wildlife and the environment, creating significant regulatory gaps [111]. As a result, when Google Wing launched its delivery trials, CASA was in no position to address urgent community concerns about broader risks and associated responsibilities.

The ACT Government first became involved with Google Wing in a regulatory capacity between July 2017 and February 2018, when the firm made several applications to license Government-owned land as launch sites for its trials in Royalla [116] and Bonython [117,118]. Each license was granted, but behind the scenes, the Bonython application kicked off a constitutional ‘football’, as ACT Government actors struggled to determine territorial jurisdiction over an activity that involved aircraft, and therefore attracted Commonwealth jurisdiction [119]. Exchanges between the ACT Government and CASA reveal that the core uncertainty was over who was responsible for managing the ‘local community impacts of delivery drones’, i.e. noise, visual pollution, disturbances to wildlife, etc. [120–122], all of which are familiar to local governments in other arenas. The story of Wing in the ACT is really a story of how addressing the multiple effects of drones on communities went from being a clear governmental priority for ‘long term policy development’ and a ‘comprehensive regulatory framework’, as the Government stated in February 2018 [91,123], to being kicked well into the long grass. How did this happen?

An illuminating marker of the ACT’s initial position is from January 2018, six months prior to the commencement of the Bonython trial, when the ACT Environment, Planning and Sustainable Development Directorate (the body responsible for approving the land use licenses) prepared an internal ‘Risk Management Assessment’, listing a relatively comprehensive set of potential risks of Wing’s operations in the ACT [124]. Recognizing that these risks all engage existing government expertise and authority, responsibilities were allocated between the ACT Government and federal partners (as indicated in square brackets):

‘(1) Noise [joint] … (2) Privacy concerns [joint] … (3) Impact on domestic pets [ACT] … (4) Impact on wildlife [ACT] … (5) Loss of amenity [ACT] … (6) Cyber security [joint] … (7) Sensitive locations [joint] … (8) Distraction risk to drivers, cyclists, and pedestrians [joint] … (9) Safety [joint] … (10) Land use concerns [ACT] … (11) Trespass [joint] … (12) ACT Government agency objections [ACT] … (13) Fire ignition [joint] … (14) Negligence [joint] … (15) Reputational damage [ACT].’ [124]

This list makes evident that many of these priorities could be actioned by the ACT alone (e.g. wildlife, amenity, land use), while others required negotiation. However, our review of the documentary record—which shows inaction on each of these matters, accompanied by a clear shift in focus towards Wing’s business and operational interests—reveals that the ACT Government’s initial political will to identify and navigate areas of responsibility was steadily chipped away over the ensuing months. Already in February 2018, we started to see the embrace of inevitability narratives, as in the following extract from a ministerial brief provided by the ACT Chief Minister, Treasury and Economic Development Directorate:

‘The development of a more specific regulatory framework will need to be assessed alongside the issues of commercial viability for drone operating businesses as well as the ACT’s reputation as a jurisdiction that welcomes innovation and technological pioneering.’ [119]

In August 2018, one month into the Bonython trial, the prospect of a comprehensive, long-term policy and regulatory approach was quietly replaced by a plan to develop ‘a light touch legislative framework for drones in the ACT’, through a newly established cross-Government working group [125]. The working group tabled a range of operational and governance issues, including the significance of community perceptions and the need to respond to complaints and concerns, but it appears these were never seriously advanced [126].

By February 2019, there had been a remarkable 180 degree shift in the ACT Government’s risk focus, away from everything highlighted in the January 2018 risk assessment, and into a new concern—protecting Google Wing’s ability to operate through minimal regulation, if not total corporate deference. This was expressed in the ACT Government’s February 2019 submission to the ACT Parliamentary Inquiry [37], launched in response to community pressure from Bonython residents, by the suggestion that the Government had no regulatory responsibilities beyond providing Wing ‘business support in the form of temporary licenses to access unleased land’ [87]. This was bolstered by the position of the key ACT planning minister:

‘Minister Gentleman is supportive of possible conditions on Wing, but wants to ensure they are broadly similar to restrictions that may be placed on other businesses or operations, so that Wing’s operations are not unduly limited. In other words, he’d like the conditions not to be overly prescriptive and restrictive.’ [127]

Internal memos from the ACT Government in November 2018 reveal antipathy to the Parliamentary Inquiry, warning of ‘risk to government if the Committee [leading the Inquiry] recommends heavy handed regulation’ [128]. The specific ‘risk’ in issue appears to be public scrutiny, via the Inquiry, of the Government’s handling of the drone delivery trials, given the Inquiry’s terms of reference included ‘the extent of regulatory oversight of drone technology at various levels of government including but not limited to … local authorities’ [129]. The Government’s about-face reflects a strategic avoidance of regulatory responsibility, and a desire to characterize Google Wing as merely another business. This feat was achieved first, by reliance on jurisdiction, and second, by reliance on the vehicle of a ‘trial’. Neither justification withstands scrutiny.

First, the ACT Government asserted that ‘the ACT Legislative Assembly does not have the legal ability to provide a comprehensive, targeted regulatory response to drone delivery’ [87]. The sleight of hand here is that because *some* aspects of drones fall outside exclusive ACT jurisdiction (in particular, air navigation and safety, which falls under the Commonwealth head of power [130]), there is a suggestion that *everything* does. Of course, this is not accurate. Yet during Inquiry hearings, when asked about ‘things that the government can potentially do [to regulate drones] and what the limits to those might be’ [131], the ACT Deputy Chief Solicitor responded evasively, erasing the prior work that had been done within the ACT Government:

‘I would not say that we have necessarily identified what you could do … As a developing area of law, it will develop as we go along. I do not think I could say that we have identified any areas we think absolutely are areas.’ [131]

This position neatly matches Google Wing’s preferred regulatory approach, as characterized by an ACT Government officer writing in November 2018: ‘Google is not thrilled at the idea of sub-national regulation—they strongly prefer national approaches, understandably’ [132].

Second, the ACT Government, primarily in public statements made by key ministers, seized on the notion of facilitating ‘trials’ [133], as an ‘opportunity to learn about challenges and opportunities presented by emerging delivery drone technology’ [99,107]. They cautioned that ‘to prematurely recommend regulatory measures, particularly in the public domain, could negate the trial’s effectiveness’ [119]. This position is also dubious. Trials—particularly government-sanctioned trials—necessitate assessment and review. There were no agreed parameters or indicators to evaluate the relative successes or failures of the Bonython trial, and ‘no forward plan of conducting an evaluation’ [128]. ACT officers’ reliance on the tenuous framing of a trial also persisted after they knew Google Wing was securing a site for ongoing operations in the north of Canberra [87].

Overall, we conclude that the ACT Government’s hands-off approach and failure to address recognized regulatory gaps was dictated by its commitment to enabling Google Wing’s business model, through an embrace of inevitability narratives of *necessity* (economic development), *desire* (innovation and leadership) and *presumption* (drones will operate, and their expansion must not be impeded; therefore, they will be treated like any other business).

### (b) Replication and political legitimization of corporate narratives

The ACT’s deference to corporate narratives also defined its community engagement and its public rationalization of the trials. Initially, in October 2017, various community engagement mechanisms were considered, with the ACT Government cautious that treating the trials as a form of consultation might elicit critique of the Government’s ‘permissive attitude’ [91]. By December 2017, the Government had resolved to ‘be slightly at arm’s length’ [134], in effect relinquishing its direct duties to affected communities by allocating them to Google Wing. As a result, the ACT Government maintained no publicly available details on drone operations, such as operating hours, flight frequency levels and relevant laws, nor did it provide a portal for feedback for residents [135]. When pressed by aggrieved residents on the lack of communication and transparency on the Bonython trial and the Government’s involvement, Chief Minister Barr adopted a stance that would be repeated throughout the Government’s dealings with constituents: ‘the ACT Government does not intend on undertaking community engagement on behalf of Project Wing. It is not usual for the ACT Government to undertake community engagement on behalf of a private company’ [107].

At the same time, the ACT Government’s communications bore striking resemblance to Google Wing’s talking points, from replicating Wing’s updated mission—‘to increase access to goods, reduce traffic congestion in cities, and help ease the CO2 emissions attributable to the transportation of goods’ [12,136]—to reinforcing Wing’s techno-solutionism in response to significant complaints that had arisen over noise (i.e. quieter drones) and privacy (i.e. low-resolution video) [136]. Beyond direct amplification of Google Wing talking points, the ACT Government’s uncritical promulgation of corporate narratives of inevitability is best demonstrated in three areas: (i) economic and environmental claims, (ii) impacts on birds and wildlife, and (iii) noise.

Google Wing and the ACT Government’s claims around the economic and environmental benefits of drone delivery were based on a 2018 report commissioned by Google Wing from consultancy firm AlphaBeta [100], which the ACT acknowledged ‘significantly helped inform the ACT Government’s position on drones’ [137]. The factual validity of the report’s projections is highly contestable, owing to the conflation of the addressable market for food delivery ($AU0.26 billion over 20 years) with military and industrial applications ($AU5.5 billion over the same period) that the report does not acknowledge, as well as the erasure of the logistics supply chain that underpins both drones and their payloads [71]. The degree to which road-based traffic is replaced by drones in practice is also not tested, and the report is premised on a narrative of presumption, that is, of vastly increased drone operations. The uncritical government acceptance and repetition of such clearly partisan information reflects the consequences of internalized narratives of inevitability.

On the vital question of detrimental effects of delivery drones on wildlife and the environment, the ACT Government relied on Wing’s narrative, even against the precaution of internal ACT Government experts. Wildlife and ecological damage caused by drone operations were among the many risks identified in the January 2018 risk assessment and as part of the terms of reference of the 2019 Parliamentary Inquiry [124]. These were also key points of concern for residents during the Bonython trial, with anecdotal reports of reduced sightings of native birds and disruptions to birds and other wildlife through physical interactions with drones [135,138–140]. The ACT Government’s submission to the Parliamentary Inquiry advised that it had raised this issue with Wing as part of issuing a land use license and, in response, Wing had engaged a research consultant whose findings were shared with the Government [87]. What the submission omitted was the response of the ACT Government’s own ecologists to Wing’s research, concluding that its scope and recommendations provided an *insufficient base* for the trial in the absence of any further mitigation measures [141]. Yet after this point, the ACT continually deferred responsibility to Wing to track effects on birds and wildlife, with the submission concluding: ‘The ACT Government is pleased Wing has undertaken to contribute to the development of research that contributes to a growing body of knowledge on implications of drone technology’ [87].

Even in the case of noise pollution, which was the ‘single largest source of negative feedback’ during the Bonython trial [135], the ACT Government failed to critically appraise the merits of information provided by Google Wing, instead praising the company’s engagement with communities and regulators and the technical fixes that had been applied to reduce drone noise. Bonython residents gave vivid descriptions of the level and pitch of noise as drones flew over homes and hovered for dispatch [135], leading to detrimental physical and mental health outcomes and the loss of overall amenity, with many residents feeling forced to physically leave their homes for extended periods of time to escape the noise [142]. Despite this, the ACT Government relied solely on measurements provided by Google Wing about the decibel levels of its drones, to conclude that they were ‘equivalent to or quieter than other activities that are part of the urban/suburban soundscape’ (e.g. lawnmowers) [87], and that they therefore complied with local noise standards. While the lack of independent measurement and validation of noise levels was consistent with the governmental deference to corporate narratives, it became a key point of cleavage used by BAD [131], as discussed next.

## 4. The people who first lived under delivery drones

This section details one community’s response to narratives of inevitability pushed by Google Wing and governments in the hope of securing ‘community tolerance’, i.e. acquiescence to changed skyscapes. It focuses on the actions of a small but mighty community group that formed during the Wing trial in Bonython, providing an account of how BAD turned the logic of inevitability inside out—and, in so doing, revealed a set of success factors for resistance.

### (a) Strategies of resistance: BAD

In August 2018, within a month of Google Wing’s launch in Bonython, the community action group BAD had formed [138]. The group started in response to the local Neighbourhood Watch area coordinator being inundated by emails and calls from distressed residents concerned about drones suddenly appearing in the sky, and seeking information about what was happening [143]. By September, the convening Committee (composed of a handful of dedicated and resourceful community members from complementary professional backgrounds, including a retired aviation law expert and a fastidious former public servant) had set up a Facebook page [144], website [145] and regular email newsletters (‘bulletins’), to share information and document community concerns among their growing membership [146]. BAD’s central goal was ‘to raise awareness of the negative impact of the drone delivery service on residents, pets and bird life in Bonython’, and ‘to stop the drones and their intrusiveness on the peace and quiet of [the] suburb’ [146].

BAD’s opposition coalesced on two fronts: ‘negative impacts’ and governance failures. Many Bonython residents were outraged over the effects of drones on the community, local wildlife and the environment. The leading point of community discontent was noise, and the harm it was causing to residents’ mental and physical health, social cohesion and the amenity of the neighbourhood [138,143]. Also significant was privacy, given the repeated overflight of drones above residences and backyards, and the corresponding lack of information and assurances regarding potential collection of private and sensitive information [138,143]. Another source of community outrage was physical safety risk. Despite the insistence of Wing and CASA that the Bonython trial involved no reportable safety incidents [88,147], BAD collected residents’ reports of unsafe drone operations, including unplanned landings, dropped payloads, drones flying close to car traffic and birds attacking and forcing down drones [138,143]. Having identified these and other concrete risks and harms, the lack of adequate mitigations through law and governance became a major aspect of BAD’s cause.

All these issues were underpinned and aggravated by what BAD identified as severe governance failures. Indeed, it was the absence of transparency, consultation, feedback and review mechanisms around the Bonython trial that galvanized the community in the first place. Unable to determine even basic details such as the trial’s duration, or which government body was responsible, BAD concluded that ‘there were no arrangements in place to actively coordinate, monitor, govern, report about or assess the trial’ [143]. Soon it became obvious to the community that the absence of effective oversight was, at least in part, a product of the regulatory gaps discussed in §3, as illustrated by the following complaint of a resident to Access Canberra (the public interface for government services):

‘Since you have referred me to CASA for my complaint about drone noise, they have responded, after six weeks, that they do not deal with noise complaints. They do not deal with environmental issues. They do not deal with noise related health issues. They have referred my complaints about this drone operation back to the ACT Government. Please explain how this operation has managed to proceed this far without any monitoring by govt agencies as regards noise, environmental impact or health related issues. Please explain why no protocol for such a trial was put in place prior to its commencement.’ [148]

Between August 2018 and February 2019, the BAD Committee ran an impressive grassroots campaign to raise awareness and address residents’ concerns. This involved the lobbying of federal and local members of parliament [149–151], media engagement with local, national and international news outlets [39,149–151], submission of freedom of information requests to relevant government departments and agencies [72–80], outreach to other community groups, such as the Tuggeranong and the Gungahlin Community Councils [152], and face-to-face surveys within the suburb to better understand residents’ concerns.

The concerted lobbying efforts of BAD were directly responsible for calls for an ACT Parliamentary Inquiry [129,150]. At the same time, and under pressure from Bonython residents, federal member for Canberra Gai Brodtmann MP sought at independent review with the Australian Parliament [151]. On 2 November 2018, the ACT Legislative Assembly referred to the Standing Committee on Economic Development and Tourism an Inquiry into Drone Delivery Systems in the ACT. The Parliamentary Inquiry’s terms of reference included:

‘(1) the decision to base the trials of the technology in the ACT and surround[s] … (2) the economic impact of drone delivery technology being tested in the ACT … (3) the extent of regulatory oversight of drone technology at various levels of government … (4) the extent of any environmental impact as a result of trialling drone delivery technology … (5) ways to improve the use of drone delivery technology within the ACT … (6) any other relevant matter … and (7) information privacy.’ [129]

In late November 2018, ACT Legislative Assembly Speaker Joy Burch MLA lodged with the Assembly a petition on behalf of 1043 Bonython residents to ‘cease Project Wing drone delivery trial in Bonython ACT’ [153]. Signatures had been collected via BAD volunteers, who doorknocked extensively in the neighbourhood. BAD found that 80% of residents they engaged with were opposed to the trial—a very different picture from the one Wing tried to paint in the Inquiry, noting that only 40% of the feedback it had received was negative [88]. This contrast illustrates the difference between proactive engagement, by surveying the general population, and passive engagement, via collection of feedback and complaints.

### (b) Resisting inevitability by centring communities

BAD’s strategy involved the systematic resistance of each elaborate layer of Google Wing’s conduct and narrative, as reinforced and legitimized by the ACT Government. This started with raising the stakes from community engagement and awareness to community *assent and approval*. Bonython residents disapproved strongly of the lack of prior consultation about the nature, timing and potential detrimental effects of the trial [138,143]. They felt that the ACT Government had failed to secure the residents’ agreement to host the trial within their suburb. They rejected Wing’s outreach activities as meeting the threshold of ‘consultation’, and denounced the Government’s referral of community members to Wing, both for information and to provide feedback:

‘During the trial period the primary point of contact for any issues was the proponent of the service, i.e. Project Wing. That is a completely unacceptable situation. [I]t is completely unacceptable for the proponent and operator of the service to be responsible for providing the necessary feedback and controls. It just fails the common sense test.’ [143]

BAD condemned the trial as an experiment imposed without their consent: ‘Bonython residents feel like they have been guinea pigs in a done-deal experiment between Project Wing and the ACT government’ [143]. Through statements such as this, community members challenged the disempowerment they experienced by being presented with delivery drones as a *fait accompli* and worked to reclaim their agency.

Second, Bonython residents refused to accept the political enablement of Google Wing discussed in §3, by consistently criticizing the absence of adequate governance and oversight, and questioning the source of the problem: the ACT Government’s choice to align with Google Wing, and to back the firm’s business interests over regulatory responsibilities. The failure to deal with regulatory gaps that emerged from this corporate deference was particularly pronounced in relation to noise:

‘I completely do not accept that the ACT government can say, ‘I’m sorry, that is CASA’s responsibility’. You need to be advocates to get good outcomes for the ACT residents, and if that requires you to sit all of the stakeholders I listed before around a table and have it out and agree that if there is a noise issue it is no good that the EPA ACT says, ‘No, I’m sorry, we don’t do aircraft noise’, and you go to Airservices Australia and they say, ‘Oh, I’m sorry, we only do piloted aircraft, not unmanned aircraft’, hang on, there is still a noise problem, and that noise problem is a place-related issue, and place is the ACT. Therefore, it is an ACT government problem.’ [143]

BAD was explicit in calling out the ACT Government’s deference to Wing. The Government’s response to many aggrieved Bonython residents seeking to discuss their substantive complaints was to emphasize the novelty of Google Wing’s business model, discounting community members’ experiences merely as information that ‘will inform the ongoing development of the business model’ [142]. Residents were perplexed by the Government’s prioritization of Wing as the operator over its constituents:

‘Are the ACT Government and Project Wing one and the same? The development of their ‘business model’ was more important than residents’ quality of life for 6 months.’ [142]

### (c) How to dissect an inevitability narrative

#### (i) Resist technical solutions to political problems

During the ACT Parliamentary Inquiry, BAD representatives successfully resisted narratives of *presumption* and *necessity* by rejecting technical solutions and adjustments (drone modifications, operational restrictions and legal updates) to substantial political problems. They skilfully reoriented questions framed as *how* to accommodate drones, back into questions of *whether* to do so in the first place. An excellent illustration is BAD Committee Convenor Nev Sheather’s response to the question from the Inquiry Chair, ‘Even if these issues are addressed, do you see it getting to a point where you would see the community supporting it: if it were quiet, if there were legislation around privacy, and if there were an assurance that the safety issues were addressed?’

‘No … [T]he trial in Bonython was with a limited number of flights, and it sent people bonkers. The proposal from Wing is to incredibly increase the number of flights … If some of the aspects such as noise were improved, this would be cancelled out by the incredible number of drones that they propose to fly.’ [143]

#### (ii) Burst bad analogies and reset the stakes

Bonython residents also rejected the rhetorical move of conflating drone delivery of convenience items within a suburban setting with emergency items in regional or remote places:

‘We also question the real purpose of having drone deliveries for minor things such as coffee and small pharmaceuticals.’ [143]

‘Having a toasty, muffin, Mexican food or coffee delivered by drone is not something Canberrans really need.’ [142]

In distinguishing different applications, the community challenged the Government to focus on when drone delivery might and might not be in the public interest, not on the mere maturation of the technology to have such a capability: ‘We are not an anti-drone group. We are anti noisy, horrible, invasive delivery drones’ [143].

Finally, BAD residents drew attention to Google Wing’s drone trial as the canary in the coalmine for how the skies above our heads are being reset by commercial interests. Google describes its drone program as the next great era of highway-building—a program to create a new layer of city infrastructure:

‘If our road traffic management systems hadn’t evolved since 1900, very few people would be driving today and relatively new use cases – like ridesharing and autonomous vehicles – would never come to fruition. Similarly, without an iterative and open approach to [Uncrewed Traffic Management], we fear a future where the sky is only accessible by a select few – severely limiting industry growth and innovation.’ [25]

BAD recognized the foundational shift that was being ushered in, and that it was taking place without the consultation or consent of residents, calling on the ACT to ask the question: *Do we want to sell off the sky above our heads, and the peace and amenity that comes with it?*

‘Do we want our skies and head space to become polluted with this constant noise? Is it really what the ACT Government wants, and the Canberra people want … the peace of all Canberra is lost to this insidious, continual noise? … Can my complaint be seriously looked at as I think this is a time, before it’s too late, to protect Canberra, its environments and its people from this pollution.’ [154]

‘Are we expected to sacrifice our whole way of life to benefit a foreign drone company? The unique character of our nature filled city will cease to exist. I have lived this reality. It is a nightmare.’ [142]

#### (iii) Puncture inevitability itself

Perhaps most remarkably, BAD explicitly rejected the narratives of technological inevitability that had allowed for Wing’s trial to proceed in the manner that it did. The Bonython community demonstrated that they had not only identified the logic of inevitability as foundational to Wing’s ambition and the ACT Government’s enablement, but they also ably rebuffed it as unfounded:

‘Just because a new technology is available, that is no reason to bring it in. You have to weigh up whether it is a disruptive technology, like these drones are, or good technology.’ [143]

‘Just because a sound is new is not a reason that people should have to adapt to it.’ [155]

‘[I]t was a farce of a trial, set up to deliver a predetermined outcome.’ [156]

‘It was a fait accompli and residents had no say in it.’ [143]

While each of these statements is a stirring call to resistance, not all could see inevitability narratives at work. Indeed, as one resident submitted: ‘Overall I feel that drones are a sign-of the times, and the inevitable’ [157].

## 5. Lessons, Logan and blue skies

One motivation for setting out BAD’s case against Google’s drones in Australia is that invigorating stories of tech resistance rarely occasion a victory lap. As this section details, the aftermath of resistance is often a quiet fade-out. Resistance is also an evolutionary game. Google Wing is what it is today because of what happened in Canberra—but so too is every community stronger, for the people who first lived under the drones. This section concludes with the round-out of Wing in Australia, and what we can learn and take forward.

### (a) The aftermath of resistance

Google Wing’s first residential drone trial ended in Bonython in early February 2019, a few weeks prior to the planned end of its license [138]—an indication, naturally never conceded, that this suburb had got the better of Wing. By early April 2019, within a month of the intense hearing days of the ACT’s Parliamentary Inquiry, Wing announced that it had commenced commercial operations in select suburbs of Gungahlin in the north of Canberra, this time operating from a privately owned site in Mitchell [99]. That Wing was allowed to expand within the ACT [87,88], in the midst of a Parliamentary Inquiry intensely scrutinizing its behavior, was astounding to many in the community, including the Gungahlin Community Council [143,152].

Based on 151 written submissions and three full days of public hearings, the Standing Committee on Economic Development and Tourism handed down its report from the Parliamentary Inquiry in July 2019. Despite the wealth of evidence that had been detailed about the serious shortcomings of both the ACT Government and Wing, the Committee’s recommendations were very modest. It suggested increased information sharing with the public through Access Canberra, as well as inter-governmental engagement between the ACT Government and federal agencies on privacy in particular and regulating drones in general. The most concrete recommendation was for the ACT Government and Wing to jointly establish an independent comparative study of wildlife numbers and behavior prior to and following commencement of drone delivery operations [135], which at the time of writing has not been produced. The ACT Government responded in support of the recommendations, while maintaining its overall optimism about the potential of drone delivery to provide economic benefits, and using the opportunity to reinforce its view that drone regulation was a matter for the Commonwealth [158]. This was reflected in the ACT’s subsequent engagement with the federal Department of Infrastructure as part of its review of drone noise regulations [137]. This federal review was a direct consequence of BAD making the detailed legal case that the Department of Infrastructure had erroneously interpreted drones to be exempt from Aircraft Noise Regulations that the Department was responsible for administering [159]. This led the Department to reconsider and ultimately change its previous position, before reviewing the ‘appropriate scope and breadth of future noise regulation’ [41].

It is noteworthy that the ACT Government’s January 2018 risk assessment had flagged the risk of ‘reputational damage’ from Wing’s drone trials and that, as transpired to be true, a ‘catastrophic consequence’ of reputational damage would be an ‘Assembly inquiry or Commission of inquiry or adverse national media’ [124]. Nevertheless, the Government watched on as Wing maintained its operations in Gungahlin for over four years. New community members and old BAD allies continued to resist the intrusiveness of delivery drones and their detrimental effects on amenity, wellbeing and wildlife, appealing directly to ACT politicians [160] and the media [40]. In August 2023, Wing quietly announced that it would cease its operations in the ACT, stating that it had ‘shifted [its] operating model’— rather than operate out of its own warehouses (as in Mitchell), it was co-locating at large shopping centres, and it had not been able to identify a suitable shopping centre in Canberra for that purpose [42]. Instead, Wing had intensified its expansion in Queensland.

### (b) Political enablement revisited: Logan

Google Wing launched in the Queensland cities of Logan in September 2019 [161], the Gold Coast in October 2022 [162] and Ipswich in April 2023 [163,164]. Each year between 2020 and at least 2023, Logan was Wing’s biggest operating site. The tale of the company’s entrance into the city is now a familiar one of political enablement, soaked in inevitability rhetoric. Logan City Council (LCC) was approached by Wing in early 2019, when the company was on the hunt for its next site. LCC conceived of its role as limited to the approval of a development application for an industrial site Wing was leasing for its warehouse and operations, along with the assessment of potential noise effects under the Logan Planning Scheme noise criteria [165]. Like the ACT Government, LCC viewed the interest of Google Wing as evidence of its growing reputation as ‘an innovative, dynamic city of the future’ [166]. Wing’s presence in Logan has been touted as proof that Logan is worthy of investment by international companies, with high hopes of local job creation [167,168]. This sentiment aligns with the Queensland Government’s aim to make the state the capital of drone technology in Australia [33]. Queensland Government actors praised the launch of Wing’s operations in Logan as ‘another feather in Queensland’s cap’ and ‘a huge vote of confidence in our community’ [169].

Logan and Canberra are very different cities in geography, history and demographics. Logan is the tenth largest local government area in Australia, with Queensland’s fastest area growth rate [170]. It does not carry Canberra’s storied history of politics, community organizing and retired public servants. Public resistance to Google Wing in Logan has remained sporadic and on an individual level [166]. But the absence of organized and public opposition should not be interpreted to imply approval among the residents of Logan, any more than it would in Canberra or the next city chosen by Google. The quirk that the ACT Government is simultaneously both a territory and local government may well have fostered a more robust participatory governance layer, in the form of unofficial community councils and similar organizations, for the highly localized fight that food delivery drones present. On a more fundamental level, though, the success of BAD’s campaign is attributable to the personal skills, resources and resilience of a small number of people that united over a shared cause.

While the case of Bonython demonstrates the possibility of public resistance, negating the logic of technological inevitability, it should not be equated with a responsibility to do so. On the contrary, the lack of comparable levels of organized grassroots resistance in Logan suggests that the occurrence and success of public resistance are highly dependent on individual circumstances, the presence or absence of which should not be taken as indicating actual approval, nor as replacing adequate governance mechanisms.

### (c) ‘Just because a sound is new’

The story of Bonython vs. Google Wing presents a fully contained case study of public resistance to private ambition. It also gives us a template for dissecting and puncturing the logic of inevitability that permeates technology. The first step is to expose the pattern: the presentation, replication and legitimization of technological developments as natural, ineluctable forces, foreclosing deliberation and refusal—even when those developments are the entirely foreign intrusion of burrito-delivering drones, streaking new tollways and creating security havoc in free blue skies. The second is to resist inevitability’s self-fulfilling logic that takes silence and resignation as beacons of acceptance and approval—just because a sound is new, it does not follow that we must adapt.

The simple fact that a community resisted a technology for which ubiquity had been proclaimed, assumed and accepted falsifies the logic of inevitability. BAD is a crucial reminder that narratives of technological inevitability are no more than stories, designed to disempower communities and governments from actively designing and demanding the futures we want and need [81].

Delivery drones are a powerful case study for participatory cities because of how viscerally they embody and forecast the risks and detrimental effects of asymmetric power between companies, governments and citizens, at the crossroads of tech expansion. As vividly illustrated in the accounts of Bonython residents, drones are highly visible and audible [143,156]. As opposed to other, less tangible technologies, drones force a direct confrontation with the changes they provoke in society and the environment, including that which we hold in common. Tech expansion for commerce and convenience into the vast public commons of the sky presents an urgent challenge for digital governance and participatory cities. The legacy of BAD is that we all have agency in the fight.

## Data Availability

No additional data has been received for this article.
